# Supplementary material for: Metabolomic Profiling in Neuromyelitis Optica Spectrum Disorder Biomarker Discovery
Source: Metabolites. 2020 Sep 18;10(9):374. doi: 10.3390/metabo10090374 (PMC7570337; doi:10.3390/metabo10090374)
Supplement: Supplementary file 1 [file metabolites-10-00374-s001.zip › Supplementary/Supplementary Table 1_NMOSD Manuscript_McKarns_10.07.19.pdf]

SupplementaryTable 1. Altered Protein and Lipoprotein Outcomes in NMOSD Proteomic Studies.

Endpoint	Shift	Biopsy	NMOSD Cohort		Disease State at Biopsy[a]		AQP4-Ab Serology		Control	Method[c]	Ref.
			Female	Male	Relapse	Remission	+	-			
Actin-α1	↑	CSF[d]	8[e]	2[e]	NR	NR	NR	NR	12 LETM[f,g]	HR LTQ-Orbitrap MS	(184)
AEBP1	↓	CSF[d]	8[e]	2[e]	NR	NR	NR	NR	12 LETM[f,g]	HR LTQ-Orbitrap MS	(184)
α1-β glycoprotein	↓	CSF	6	3	9	–	NR	NR	9 OND	2-DE & MALDI-TOF-MS	(187)
α1-acid glycoprotein	↓	CSF[d]	8[e]	2[e]	NR	NR	NR	NR	12 LETM[f,g]	HR LTQ-Orbitrap MS	(184)
α1-antichymotrypsin	↓	CSF[d]	8[e]	2[e]	NR	NR	NR	NR	12 LETM[f,g]	HR LTQ-Orbitrap MS	(184)
α1-antitrypsin	↓	CSF[d]	8[e]	2[e]	NR	NR	NR	NR	12 LETM[f,g]	HR LTQ-Orbitrap MS	(184)
α2-macroglobulin	↑	CSF[d]	8[e]	2[e]	NR	NR	NR	NR	12 LETM[f,g]	HR LTQ-Orbitrap MS	(184)
Albumin	↓	CSF[d]	8[e]	2[e]	NR	NR	NR	NR	12 LETM[f,g]	HR LTQ-Orbitrap MS	(184)
Amyloid-like protein 1	↓	CSF[d]	8[e]	2[e]	NR	NR	NR	NR	12 LETM[f,g]	HR LTQ-Orbitrap MS	(184)
Angiotensinogen	↓	CSF[d]	8[e]	2[e]	NR	NR	NR	NR	12 LETM[f,g]	HR LTQ-Orbitrap MS	(184)
Antithrombin III	↓	CSF[d]	8[e]	2[e]	NR	NR	NR	NR	12 LETM[f,g]	HR LTQ-Orbitrap MS	(184)
Apolipoprotein A-I	↓	CSF[d]	8[e]	2[e]	NR	NR	NR	NR	12 LETM[f,g]	HR LTQ-Orbitrap MS	(184)
	↓	CSF	4	2	6	–	NR	NR	6 OND	2-DE & MALDI-TOF-MS	(188)
Apolipoprotein A-II	↓	CSF[d]	8[e]	2[e]	NR	NR	NR	NR	12 LETM[f,g]	HR LTQ-Orbitrap MS	(184)
Apolipoprotein A-IV	↓	CSF[d]	8[e]	2[e]	NR	NR	NR	NR	12 LETM[f,g]	HR LTQ-Orbitrap MS	(184)
	↓	CSF	6	3	9	–	NR	NR	9 OND	2-DE & MALDI-TOF-MS	(187)
Apolipoprotein B-100	↑	CSF[d]	8[e]	2[e]	NR	NR	NR	NR	12 LETM[f,g]	HR LTQ-Orbitrap MS	(184)
Apolipoprotein D	↓	CSF[d]	8[e]	2[e]	NR	NR	NR	NR	12 LETM[f,g]	HR LTQ-Orbitrap MS	(184)
Apolipoprotein E	↓	CSF[d]	8[e]	2[e]	NR	NR	NR	NR	12 LETM[f,g]	HR LTQ-Orbitrap MS	(184)
	↓	CSF	6	3	9	–	NR	NR	9 OND	2-DE & MALDI-TOF-MS	(187)
	↓	CSF	4	2	6	–	NR	NR	6 OND	2-DE & MALDI-TOF-MS	(188)
Attractin	↓	Urine	28	4	–	32	32	–	31 HC	LC-MS/MS	(189)
β-Ala-His Dipeptidase	↓	CSF[d]	8[e]	2[e]	NR	NR	NR	NR	12 LETM[f,g]	HR LTQ-Orbitrap MS	(184)
β-Mannosidase	↓	Urine	28	4	–	32	32	–	31 HC	LC-MS/MS	(189)
Bile salt-activated lipase	↓	Urine	28	4	–	32	32	–	31 HC	LC-MS/MS	(189)
C1 esterase inhibitor	↓	CSF[d]	8[e]	2[e]	NR	NR	NR	NR	12 LETM[f,g]	HR LTQ-Orbitrap MS	(184)
C4BPα	↑	CSF[d]	8[e]	2[e]	NR	NR	NR	NR	12 LETM[f,g]	HR LTQ-Orbitrap MS	(184)
CACNA2D2	↓	CSF[d]	8[e]	2[e]	NR	NR	NR	NR	12 LETM[f,g]	HR LTQ-Orbitrap MS	(184)
Cadherin-2	↓	Urine	28	4	–	32	32	–	46 RRMS, 31 HC	LC-MS/MS	(189)
Cadherin-19	↓	Urine	28	4	–	32	32	–	31 HC	LC-MS/MS	(189)
Calsyntenin-1	↓	CSF[d]	8[e]	2[e]	NR	NR	NR	NR	12 LETM[f,g]	HR LTQ-Orbitrap MS	(184)
Carboxypeptidase E	↓	CSF[d]	8[e]	2[e]	NR	NR	NR	NR	12 LETM[f,g]	HR LTQ-Orbitrap MS	(184)
Cathepsin D	↓	CSF[d]	8[e]	2[e]	NR	NR	NR	NR	12 LETM[f,g]	HR LTQ-Orbitrap MS	(184)
CD9	↓	Urine	28	4	–	32	32	–	31 HC	LC-MS/MS	(189)
CD84	↓	Urine	28	4	–	32	32	–	31 HC	LC-MS/MS	(189)
Ceruloplasmin	↑	CSF[d]	8[e]	2[e]	NR	NR	NR	NR	12 LETM[f,g]	HR LTQ-Orbitrap MS	(184)
Chitinase-3-like protein	↑	CSF[d]	8[e]	2[e]	NR	NR	NR	NR	12 LETM[f,g]	HR LTQ-Orbitrap MS	(184)
CHL1	↓	CSF[d]	8[e]	2[e]	NR	NR	NR	NR	12 LETM[f,g]	HR LTQ-Orbitrap MS	(184)
CHMP5	↓	Urine	28	4	–	32	32	–	31 HC	LC-MS/MS	(189)
Chromogranin A	↓	CSF[d]	8[e]	2[e]	NR	NR	NR	NR	12 LETM[f,g]	HR LTQ-Orbitrap MS	(184)
Clusterin	↓	CSF[d]	8[e]	2[e]	NR	NR	NR	NR	12 LETM[f,g]	HR LTQ-Orbitrap MS	(184)
Collagen α1, type I	↓	CSF[d]	8[e]	2[e]	NR	NR	NR	NR	12 LETM[f,g]	HR LTQ-Orbitrap MS	(184)
Collagen α1, type IV	↓	CSF[d]	8[e]	2[e]	NR	NR	NR	NR	12 LETM[f,g]	HR LTQ-Orbitrap MS	(184)
Collagen α1, type XVIII	↓	CSF[d]	8[e]	2[e]	NR	NR	NR	NR	12 LETM[f,g]	HR LTQ-Orbitrap MS	(184)
	↓	Urine	28	4	–	32	32	–	31 HC	LC-MS/MS	(189)
Complement C1q	↑	CSF[d]	8[e]	2[e]	NR	NR	NR	NR	12 LETM[f,g]	HR LTQ-Orbitrap MS	(184)
Complement C1r	↑	CSF[d]	8[e]	2[e]	NR	NR	NR	NR	12 LETM[f,g]	HR LTQ-Orbitrap MS	(184)
Complement C1s	↑	CSF[d]	8[e]	2[e]	NR	NR	NR	NR	12 LETM[f,g]	HR LTQ-Orbitrap MS	(184)
Complement C3	↑	CSF[d]	8[e]	2[e]	NR	NR	NR	NR	12 LETM[f,g]	HR LTQ-Orbitrap MS	(184)
Complement C4	↑	CSF[d]	8[e]	2[e]	NR	NR	NR	NR	12 LETM[f,g]	HR LTQ-Orbitrap MS	(184)
Complement C5	↑	CSF[d]	8[e]	2[e]	NR	NR	NR	NR	12 LETM[f,g]	HR LTQ-Orbitrap MS	(184)
Complement C6	↑	CSF[d]	8[e]	2[e]	NR	NR	NR	NR	12 LETM[f,g]	HR LTQ-Orbitrap MS	(184)
Complement C7	↑	CSF[d]	8[e]	2[e]	NR	NR	NR	NR	12 LETM[f,g]	HR LTQ-Orbitrap MS	(184)

Complement C8 β	↑	CSF[d]	8[e]	2[e]	NR	NR	NR	NR	12 LETM[f,g]	HR LTQ-Orbitrap MS	(184)
Complement C8 γ	↑	CSF[d]	8[e]	2[e]	NR	NR	NR	NR	12 LETM[f,g]	HR LTQ-Orbitrap MS	(184)
Complement C9	↑	CSF[d]	8[e]	2[e]	NR	NR	NR	NR	12 LETM[f,g]	HR LTQ-Orbitrap MS	(184)
Contactin-1	↓	CSF[d]	8[e]	2[e]	NR	NR	NR	NR	12 LETM[f,g]	HR LTQ-Orbitrap MS	(184)
	↓	Urine	28	4	–	32	32	–	31 HC	LC-MS/MS	(189)
Cystatin-C	↓	CSF[d]	8[e]	2[e]	NR	NR	NR	NR	12 LETM[f,g]	HR LTQ-Orbitrap MS	(184)
DKK3	↓	CSF[d]	8[e]	2[e]	NR	NR	NR	NR	12 LETM[f,g]	HR LTQ-Orbitrap MS	(184)
Factor H	↑	CSF[d]	8[e]	2[e]	NR	NR	NR	NR	12 LETM[f,g]	HR LTQ-Orbitrap MS	(184)
FAM3C	↓	CSF[d]	8[e]	2[e]	NR	NR	NR	NR	12 LETM[f,g]	HR LTQ-Orbitrap MS	(184)
FGFR1	↓	Urine	28	4	–	32	32	–	31 HC	LC-MS/MS	(189)
Fibrinogen α	↑	CSF[d]	8[e]	2[e]	NR	NR	NR	NR	12 LETM[f,g]	HR LTQ-Orbitrap MS	(184)
Fibrinogen β	↑	CSF[d]	8[e]	2[e]	NR	NR	NR	NR	12 LETM[f,g]	HR LTQ-Orbitrap MS	(184)
Fibrinogen γ	↑	CSF[d]	8[e]	2[e]	NR	NR	NR	NR	12 LETM[f,g]	HR LTQ-Orbitrap MS	(184)
	↓	CSF	6	3	9	–	NR	NR	9 OND	2-DE & MALDI -TOF-MS	(187)
Fibronectin	↓	CSF[d]	8[e]	2[e]	NR	NR	NR	NR	12 LETM[f,g]	HR LTQ-Orbitrap MS	(184)
Fibulin-1	↓	CSF[d]	8[e]	2[e]	NR	NR	NR	NR	12 LETM[f,g]	HR LTQ-Orbitrap MS	(184)
Ficolin-3	↑	CSF[d]	8[e]	2[e]	NR	NR	NR	NR	12 LETM[f,g]	HR LTQ-Orbitrap MS	(184)
Follistatin-like 1	↓	Urine	28	4	–	32	32	–	31 HC	LC-MS/MS	(189)
Frizzled-4	↓	Urine	28	4	–	32	32	–	31 HC	LC-MS/MS	(189)
GAPDH	↑	CSF[d]	8[e]	2[e]	NR	NR	NR	NR	12 LETM[f,g]	HR LTQ-Orbitrap MS	(184)
Gelsolin	↑	CSF[d]	8[e]	2[e]	NR	NR	NR	NR	12 LETM[f,g]	HR LTQ-Orbitrap MS	(184)
GFAP	↑	CSF[d]	8[e]	2[e]	NR	NR	NR	NR	12 LETM[f,g]	HR LTQ-Orbitrap MS	(184)
GnTE	↓	CSF[d]	8[e]	2[e]	NR	NR	NR	NR	12 LETM[f,g]	HR LTQ-Orbitrap MS	(184)
Haptoglobin	↑	CSF[d]	8[e]	2[e]	NR	NR	NR	NR	12 LETM[f,g]	HR LTQ-Orbitrap MS	(184)
	↑	Serum	3	3	6	–	NR	NR	6 RRMS, 6 HC	2-DE & HD-MS/MS	(185)
	↑	CSF	6	3	9	–	NR	NR	9 OND	2-DE & MALDI-TOF-MS	(187)
	↑	CSF	4	2	6	–	NR	NR	6 OND	2-DE & MALDI-TOF-MS	(188)
Haptoglobin related protein	↑	CSF[d]	8[e]	2[e]	NR	NR	NR	NR	12 LETM[f,g]	HR LTQ-Orbitrap MS	(184)
Hemoglobin	↓	CSF[d]	8[e]	2[e]	NR	NR	NR	NR	12 LETM[f,g]	HR LTQ-Orbitrap MS	(184)
HSPG	↓	CSF[d]	8[e]	2[e]	NR	NR	NR	NR	12 LETM[f,g]	HR LTQ-Orbitrap MS	(184)
ICAM-2	↓	Urine	28	4	–	32	32	–	46 RRMS, 31 HC	LC-MS/MS	(189)
IGFBP7	↓	Urine	28	4	–	32	32	–	31 HC	LC-MS/MS	(189)
IGFALS	↓	Urine	28	4	–	32	32	–	31 HC	LC-MS/MS	(189)
Ig heavy, constant α1	↑	CSF[d]	8[e]	2[e]	NR	NR	NR	NR	12 LETM[f,g]	HR LTQ-Orbitrap MS	(184)
Ig heavy, constant α2	↑	CSF[d]	8[e]	2[e]	NR	NR	NR	NR	12 LETM[f,g]	HR LTQ-Orbitrap MS	(184)
Ig heavy, variable 3	↑	CSF[d]	8[e]	2[e]	NR	NR	NR	NR	12 LETM[f,g]	HR LTQ-Orbitrap MS	(184)
Ig heavy, constant γ1	↑	CSF[d]	8[e]	2[e]	NR	NR	NR	NR	12 LETM[f,g]	HR LTQ-Orbitrap MS	(184)
Ig heavy, constant γ2	↑	CSF[d]	8[e]	2[e]	NR	NR	NR	NR	12 LETM[f,g]	HR LTQ-Orbitrap MS	(184)
Ig heavy, constant γ3	↑	CSF	6	3	9	–	NR	NR	9 OND	2-DE & MALDI-TOF-MS	(187)
	↑	Urine	28	4	–	32	32	–	46 RRMS, 31 HC	LC-MS/MS	(189)
Ig heavy, constant μ	↑	CSF[d]	8[e]	2[e]	NR	NR	NR	NR	12 LETM[f,g]	HR LTQ-Orbitrap MS	(184)
IgG F_c binding protein	↑	CSF[d]	8[e]	2[e]	NR	NR	NR	NR	12 LETM[f,g]	HR LTQ-Orbitrap MS	(184)
Ig κ, constant	↑	CSF[d]	8[e]	2[e]	NR	NR	NR	NR	12 LETM[f,g]	HR LTQ-Orbitrap MS	(184)
	↑	CSF	6	3	9	–	NR	NR	9 OND	2-DE & MALDI-TOF-MS	(187)
Ig κ, variable 1	↑	CSF[d]	8[e]	2[e]	NR	NR	NR	NR	12 LETM[f,g]	HR LTQ-Orbitrap MS	(184)
Ig κ, variable 3	↑	CSF[d]	8[e]	2[e]	NR	NR	NR	NR	12 LETM[f,g]	HR LTQ-Orbitrap MS	(184)
	↑	Urine	28	4	–	32	32	–	31 HC	LC-MS/MS	(189)
Ig κ, variable 4	↑	CSF[d]	8[e]	2[e]	NR	NR	NR	NR	12 LETM[f,g]	HR LTQ-Orbitrap MS	(184)
Ig λ	↓	Serum	3	3	6	–	NR	NR	6 RRMS	2-DE & HD-MS/MS	(185)
Ig λ, constant 2	↑	CSF[d]	8[e]	2[e]	NR	NR	NR	NR	12 LETM[f,g]	HR LTQ-Orbitrap MS	(184)
	↑	Urine	28	4	–	32	32	–	31 HC	LC-MS/MS	(189)
Ig λ-like polypeptide 5	↑	CSF[d]	8[e]	2[e]	NR	NR	NR	NR	12 LETM[f,g]	HR LTQ-Orbitrap MS	(184)
Ig λ, variable 3	↑	CSF[d]	8[e]	2[e]	NR	NR	NR	NR	12 LETM[f,g]	HR LTQ-Orbitrap MS	(184)
Integral membrane protein	↓	Urine	28	4	–	32	32	–	31 HC	LC-MS/MS	(189)
Inter-α-trypsin inhibitors	↑	CSF[d]	8[e]	2[e]	NR	NR	NR	NR	12 LETM[f,g]	HR LTQ-Orbitrap MS	(184)
Interleukin-10 receptor β	↓	Urine	28	4	–	32	32	–	31 HC	LC-MS/MS	(189)

JAML	↓	Urine	28	4	–	32	32	–	31 HC	LC-MS/MS	(189)
Kallikrein-1	↓	Urine	28	4	–	32	32	–	31 HC	LC-MS/MS	(189)
Kallistatin	↓	CSF[d]	8[e]	2[e]	NR	NR	NR	NR	12 LETM[f,g]	HR LTQ-Orbitrap MS	(184)
Keratin 1	↑	CSF	3	3	6	–	NR	NR	6 RRMS, 6 HC	2-DE & HD-MS/MS	(186)
	↓	CSF[d]	8[e]	2[e]	NR	NR	NR	NR	12 LETM[f,g]	HR LTQ-Orbitrap MS	(184)
Keratin 2E	↓	CSF[d]	8[e]	2[e]	NR	NR	NR	NR	12 LETM[f,g]	HR LTQ-Orbitrap MS	(184)
Keratin 5	↓	CSF[d]	8[e]	2[e]	NR	NR	NR	NR	12 LETM[f,g]	HR LTQ-Orbitrap MS	(184)
Keratin 6C	↓	CSF[d]	8[e]	2[e]	NR	NR	NR	NR	12 LETM[f,g]	HR LTQ-Orbitrap MS	(184)
Keratin 9	↓	CSF[d]	8[e]	2[e]	NR	NR	NR	NR	12 LETM[f,g]	HR LTQ-Orbitrap MS	(184)
	↑	CSF	3	3	6	–	NR	NR	6 HC	2-DE & HD-MS/MS	(186)
Keratin 10	↓	CSF[d]	8[e]	2[e]	NR	NR	NR	NR	12 LETM[f,g]	HR LTQ-Orbitrap MS	(184)
Keratin 83	↓	Serum	3	3	6	–	NR	NR	6 RRMS	2-DE & HD-MS/MS	(185)
L-lactate dehydrogenase B	↑	CSF[d]	8[e]	2[e]	NR	NR	NR	NR	12 LETM[f,g]	HR LTQ-Orbitrap MS	(184)
Laminin γ1	↓	Urine	28	4	–	32	32	–	31 HC	LC-MS/MS	(189)
Mimecan	↓	CSF[d]	8[e]	2[e]	NR	NR	NR	NR	12 LETM[f,g]	HR LTQ-Orbitrap MS	(184)
NCAM-1	↓	CSF[d]	8[e]	2[e]	NR	NR	NR	NR	12 LETM[f,g]	HR LTQ-Orbitrap MS	(184)
NCAM-2	↓	CSF[d]	8[e]	2[e]	NR	NR	NR	NR	12 LETM[f,g]	HR LTQ-Orbitrap MS	(184)
Neogenin	↓	CSF[d]	8[e]	2[e]	NR	NR	NR	NR	12 LETM[f,g]	HR LTQ-Orbitrap MS	(184)
Neurofascin	↓	CSF[d]	8[e]	2[e]	NR	NR	NR	NR	12 LETM[f,g]	HR LTQ-Orbitrap MS	(184)
Neurofilament	↑	CSF	6	3	9	–	NR	NR	9 OND	2-DE & MALDI-TOF-MS	(187)
	↑	CSF	4	2	6	–	NR	NR	6 OND	2-DE & MALDI-TOF-MS	(188)
Neuronal growth factor 1	↓	Urine	28	4	–	32	32	–	31 HC	LC-MS/MS	(189)
Neuronal pentraxin 1	↓	CSF[d]	8[e]	2[e]	NR	NR	NR	NR	12 LETM[f,g]	HR LTQ-Orbitrap MS	(184)
Neuronal pentraxin receptor	↓	CSF[d]	8[e]	2[e]	NR	NR	NR	NR	12 LETM[f,g]	HR LTQ-Orbitrap MS	(184)
Neurotrimin	↓	Urine	28	4	–	32	32	–	31 HC	LC-MS/MS	(189)
Nidogen-2	↓	CSF[d]	8[e]	2[e]	NR	NR	NR	NR	12 LETM[f,g]	HR LTQ-Orbitrap MS	(184)
OPCML	↓	Urine	28	4	–	32	32	–	31 HC	LC-MS/MS	(189)
PAM	↓	CSF[d]	8[e]	2[e]	NR	NR	NR	NR	12 LETM[f,g]	HR LTQ-Orbitrap MS	(184)
PEDF	↓	CSF[d]	8[e]	2[e]	NR	NR	NR	NR	12 LETM[f,g]	HR LTQ-Orbitrap MS	(184)
	↓	CSF	6	3	9	–	NR	NR	9 OND	2-DE & MALDI-TOF-MS	(187)
	↓	CSF	4	2	6	–	NR	NR	6 OND	2-DE & MALDI-TOF-MS	(188)
Plasminogen	↑	CSF[d]	8[e]	2[e]	NR	NR	NR	NR	12 LETM[f,g]	HR LTQ-Orbitrap MS	(184)
Pre-albumin	↓	CSF	3	3	6	–	NR	NR	6 RRMS	2-DE & HD-MS/MS	(186)
Pregnancy zone protein	↑	CSF[d]	8[e]	2[e]	NR	NR	NR	NR	12 LETM[f,g]	HR LTQ-Orbitrap MS	(184)
Pro-LRP1	↑	CSF[d]	8[e]	2[e]	NR	NR	NR	NR	12 LETM[f,g]	HR LTQ-Orbitrap MS	(184)
Prosaposin	↓	Urine	28	4	–	32	32	–	46 RRMS	LC-MS/MS	(189)
Prostaglandin-D H2	↓	CSF[d]	8[e]	2[e]	NR	NR	NR	NR	12 LETM[f,g]	HR LTQ-Orbitrap MS	(184)
Protocadherin-16	↓	Urine	28	4	–	32	32	–	31 HC	LC-MS/MS	(189)
PTPRS	↓	Urine	28	4	–	32	32	–	31 HC	LC-MS/MS	(189)
QPCT	↓	Urine	28	4	–	32	32	–	31 HC	LC-MS/MS	(189)
Retinol binding protein 4	↓	CSF[d]	8[e]	2[e]	NR	NR	NR	NR	12 LETM[f,g]	HR LTQ-Orbitrap MS	(184)
Secretogranin-1	↓	CSF[d]	8[e]	2[e]	NR	NR	NR	NR	12 LETM[f,g]	HR LTQ-Orbitrap MS	(184)
Serotransferrin	↓	CSF[d]	8[e]	2[e]	NR	NR	NR	NR	12 LETM[f,g]	HR LTQ-Orbitrap MS	(184)
SPARC-like protein 1	↓	CSF[d]	8[e]	2[e]	NR	NR	NR	NR	12 LETM[f,g]	HR LTQ-Orbitrap MS	(184)
Tenascin-X	↓	CSF[d]	8[e]	2[e]	NR	NR	NR	NR	12 LETM[f,g]	HR LTQ-Orbitrap MS	(184)
Transferrin	↑	CSF	3	3	6	–	NR	NR	6 HC	2-DE & HD-MS/MS	(186)
TREM 132A	↓	CSF[d]	8[e]	2[e]	NR	NR	NR	NR	12 LETM[f,g]	HR LTQ-Orbitrap MS	(184)
Transthyretin	↓	CSF[d]	8[e]	2[e]	NR	NR	NR	NR	12 LETM[f,g]	HR LTQ-Orbitrap MS	(184)
	↓	CSF	6	3	9	–	NR	NR	9 OND	2-DE & MALDI-TOF-MS	(187)
	↓	CSF	4	2	6	–	NR	NR	6 OND	2-DE & MALDI-TOF-MS	(188)
Ubiquitin	↓	Urine	28	4	–	32	32	–	31 HC	LC-MS/MS	(189)
Vitamin D binding protein	↓	CSF	6	3	9	–	NR	NR	9 OND	2-DE & MALDI-TOF-MS	(187)

2-DE, two dimensional gel electrophoresis; AEBP1, adipocyte enhancer binding protein 1; C4BPα, C4b-binding protein alpha chain; CACNA2D2, voltage-dependent calcium channel subunit alpha-2/delta-1; CHL1, neural cell adhesion molecule L1-like; CHMP5, chromatin-modifying protein 5; CSF, cerebrospinal fluid; 3DKK3, Dickkopf-related protein 3; FAM3C, family with sequence similarity 3 member C; FGFR1, fibroblast growth factor receptor 1; Gal3BP, galectin-3-binding protein; GAPDH, glyceraldehyde-3-phosphate dehydrogenase; GFAP, glial fibrillary acidic protein; GnTE, N-acetyllactosaminide beta-1,3-N-acetylglucosaminyltransferase; HC, healthy controls; HD-MS/MS, high definition tandem mass spectrometry; HR LTQ-Orbitrap MS, high resolution hybrid linear ion trap quadrupole-orbitrap mass spectroscopy; HSPG, basement membrane-specific heparin sulfate proteoglycan core protein; ICAM-2, intercellular adhesion molecule 2; IGFBP7, insulin-like growth factor-binding protein 7; IGFALS, insulin-like growth factor-binding protein complex acid labile subunit; Ig, immunoglobulin; JAML, junctional adhesion molecule-like; LC-MS, liquid chromatography-tandem mass spectrometry; LETM, longitudinally extensive transverse myelitis; MALDI-TOF-MS, matrix-assisted laser desorption ionization time of flight mass spectrometry; neuronal pentraxin receptor; NCAM, neural cell adhesion molecule 1 & 2; NMOSD, neuromyelitis optica spectrum disorder; NR, not reported; OPCML, opioid-binding protein/cell adhesion molecule; OND, other neurological disorders, e.g., migraines, tension-type headaches, drug-induced delirium, normal pressure hydrocephalus, or trigeminal neuralgia diseases; PAM, peptidyl-glycine alpha-amidating monooxygenase; PEDF, pigment epithelium-derived factor; Pro-LRP1, pro low-density lipoprotein receptor-related protein 1; PTPRS, receptor-type tyrosine-protein phosphatase S; QPCT, glutaminyl-peptide cyclotransferase; RRMS, relapsing-remitting multiple sclerosis; TREM, transmembrane protein 132.

[a]*NMOSD status at the time of biopsy sampling.*

[b]*NMOSD samples are statistically different from each comparison group.*

[c]*Method of metabolomic spectra data acquisition.*

[d]*Proteomic analysis was performed using CSF exosomes.*

[e]*Five NMOSD CSF biopsies, with the highest MCP-1 and IP-10, were pooled prior to analysis.*

[f]*Six LETM CSF biopsies, with the highest MCP-1 & IP-10, were pooled prior to analysis.*

[g]*NMOSD Patients are* $AQP4^{-}$ *and* MOG^{-}.

[h]*T2 weighted hyperintense* lesions, *>3 vertebral segments, spinal cord MRI.*
