# Supplementary material for: Metabolomic Profiling in Neuromyelitis Optica Spectrum Disorder Biomarker Discovery
Source: Metabolites. 2020 Sep 18;10(9):374. doi: 10.3390/metabo10090374 (PMC7570337; doi:10.3390/metabo10090374)
Supplement: Supplementary file 1 [file metabolites-10-00374-s001.zip › Supplementary/Supplementary Table 2_NMOSD Manuscript_McKarns_10.07.19.pdf]

Supplementary Table 2. CSF Outcomes in Targeted Immunoassay-based NMOSD Studies.

Endpoint	Shift	NMOSD Cohort		Disease State at Biopsy[a]		AQP4-Ab Serology		Treatment[b]	Control[c]	Ref.
		Fema	M	LAP	REM	+	-			
AMCase	↑	8	4	9	3	NR	NR	–	24 RRMS, 24 SPMS, 24 OIND, 24 HC	(214)
Amyloid β-40	↔	8	2	NR	NR	NR	NR	9 Received Therapy[d]	10 MS, 6 LETM[e]	(184)
Amyloid β-42	↔	8	2	NR	NR	NR	NR	9 Received Therapy[d]	10 MS, 6 LETM[e]	(184)
APRIL	↑	16	6	22	–	NR	NR	–	18 RRMS, 14 ONND	(229)
BAFF	↑	16	6	22	–	NR	NR	–	18 RRMS, 14 ONND	(229)
	↑	7	3	10	–	NR	NR	–	10 RRMS, 10 ONND	(236)
	↑	29	8	37	–	NR	NR	PDN[f]	21 RRMS, 30 ONND	(252)
Basic fibroblast growth factor	↔	8	2	NR	NR	NR	NR	9 Received Therapy[d]	10 MS, 6 LETM[e]	(184)
	↔	31	–	31	–	25	6	19 Tx[g], 15 PDN, 7 IFN-β	18 ONND	(230)
	↔	17	3	16	4	19	1	2 SP, 5 PDN, 1 IFN-β, 1 IVIG	26 RRMS, 9 PPMS, 18 OND	(232)
	↓	31	–	31	–	25	6	19 Tx[g], 15 PDN, 7 IFN-β	29 RRMS	(230)
C3a	↔	15	–	15	–	15	–	NR	15 MS, 12 OND	(223)
	↔	19	7	26	–	21	5	–	25 RRMS, 19 OND	(225)
C4a	↔	15	–	15	–	15	–	NR	15 MS, 12 OND	(223)
C5a	↑	15	–	15	–	15	–	NR	12 OND	(223)
	↑	19	7	26	–	21	5	–	19 OND	(225)
	↔	15	–	15	–	15	–	NR	15 MS	(223)
	↔	19	7	26	–	21	5	–	25 RRMS	(225)
C5b-9	↑	19	7	26	–	21	5	–	25 RRMS, 19 OND	(225)
CCL2	↔	8	2	NR	NR	NR	NR	9 Received Therapy[d]	10 MS, 6 LETM[e]	(184)
	↔	31	–	31	–	25	6	19 Tx[g], 15 PDN, 7 IFN-β	29 RRMS, 18 ONND	(230)
	↔	17	3	16	4	19	1	2 SP, 5 PDN, 1 IFN-β, 1 IVIG	26 RRMS, 9 PPMS, 18 OND	(232)
	↔	19[h]	1[h]	20	–	NR	NR	10 IFN-β or Corticosteroids	20 RRMS	(235)
	↔	NR[hk]	NR[hk]	10	–	NR	NR	–	15 RRMS,[l] 19 SCD	(235)
	↔	13	–	13	–	13	–	8 Immune Suppressed[i]	15 MS	(242)
	↔	13	–	13	–	NR	NR	–	17 MS, 15 ONND	(244)
	↔	8	1	–	9	8	1	NR	8 L-NMOSD[j], 13 RRMS	(245)
	↓	19[h]	1[h]	20	–	NR	NR	10 IFN-β or Corticosteroids	19 SCD	(235)
CCL3	↔	8	2	NR	NR	NR	NR	9 Received Therapy[d]	10 MS, 6 LETM[e]	(184)
	↔	31	–	31	–	25	6	19 Tx[g], 15 PDN, 7 IFN-β	29 RRMS, 18 ONND	(230)
	↔	17	3	16	4	19	1	2 SP, 5 PDN, 1 IFN-β, 1 IVIG	26 RRMS, 9 PPMS, 18 OND	(232)
CCL4	↑	17	3	16	4	19	1	2 SP, 5 PDN, 1 IFN-β, 1 IVIG	18 OND	(232)
	↑	19[h]	1[h]	20	–	NR	NR	10 IFN-β or Corticosteroids	19 SCD	(235)
	↑	NR[hk]	NR[hk]	10	–	NR	NR	–	19 SCD	(235)
	↔	8	2	NR	NR	NR	NR	9 Received Therapy[d]	10 MS, 6 LETM[e]	(184)
	↔	31	–	31	–	25	6	19 Tx,[g] 15 PDN, 7 IFN-β	18 ONND	(230)
	↔	17	3	16	4	19	1	2 SP, 5 PDN, 1 IFN-β, 1 IVIG	26 RRMS, 9 PPMS	(232)
	↔	19[h]	1[h]	20	–	NR	NR	10 IFN-β or Corticosteroids	20 RRMS	(235)
	↔	NR[hk]	NR[hk]	10	–	NR	NR	–	15 RRMS[l]	(235)
	↓	31	–	31	–	25	6	19 Tx[g], 15 PDN, 7 IFN-β	29 RRMS	(230)
CCL5	↔	8	2	NR	NR	NR	NR	9 Received Therapy[d]	10 MS, 6 LETM[e]	(184)
	↔	31	–	31	–	25	6	19 Tx[g], 15 PDN, 7 IFN-β	29 RRMS, 18 ONND	(230)
	↔	17	3	16	4	19	1	2 SP, 5 PDN, 1 IFN-β, 1 IVIG	26 RRMS, 9 PPMS, 18 OND	(232)
	↔	8	1	–	9	8	1	NR	8 L-NMOSD[j], 13 RRMS	(245)
CCL11	↔	8	2	NR	NR	NR	NR	9 Received Therapy[d]	10 MS, 6 LETM[e]	(184)
	↔	31	–	31	–	25	6	19 Tx[g], 15 PDN, 7 IFN-β	29 RRMS, 18 ONND	(230)
	↔	17	3	16	4	19	1	2 SP, 5 PDN, 1 IFN-β, 1 IVIG	26 RRMS, 9 PPMS, 18 OND	(232)
	↔	13	–	13	–	NR	NR	–	17 MS, 15 ONND	(244)
CCL17	↑	13	–	13	–	NR	NR	–	15 ONND	(244)
	↔	13	–	13	–	NR	NR	–	17 MS	(244)
CCL24	↑	8	4	9	3	NR	NR	–	24 RRMS, 24 SPMS, 24 OIND, 24 HC	(214)
	↑	6	2	8	–	NR	NR	–	16 RRMS, 16 SPMS, 16 HC	(66)
CCL26	↑	8	4	9	3	NR	NR	–	24 RRMS, 24 SPMS, 24 OIND, 24 HC	(214)

	↑	6	2	8	–	NR	NR	–	16 RRMS, 16 SPMS, 16 HC	(66)
CD59	↑	28	2	30	–	26	4	NR	24 ONND	(254)
	↔	28	2	30	–	26	4	NR	22 RRMS	(254)
CHI3L1	↑	8	4	9	3	NR	NR	–	24 RRMS, 24 SPMS, 24 OIND, 24 HC	(214)
ChT	↑	8	4	9	3	NR	NR	–	24 RRMS, 24 SPMS, 24 OIND, 24 HC	(214)
CXCL8	↑	31	–	31	–	25	6	19 Tx[g], 15 PDN, 7 IFN-β	29 RRMS, 18 ONND	(230)
	↑	17	3	16	4	19	1	2 SP, 5 PDN, 1 IFN-β, 1 IVIG	26 RRMS, 9 PPMS, 18 OND	(232)
	↑	19[h]	1[h]	20	–	NR	NR	10 IFN-β or Corticosteroids	20 RRMS, 19 SCD	(235)
	↑	NR[hk]	NR[hk]	10	–	NR	NR	–	19 SCD	(235)
	↑	13	–	13	–	13	–	8 Immune Suppressed[i]	15 MS	(242)
	↔	8	2	NR	NR	NR	NR	9 Received Therapy[d]	10 MS, 6 LETM[e]	(184)
	↔	NR[hk]	NR[hk]	10	–	NR	NR	–	15 RRMS[l]	(235)
	↔	8	1	–	9	8	1	NR	8 L-NMOSD[j], 13 RRMS	(245)
CXCL9	↔	8	1	–	9	8	1	NR	8 L-NMOSD[j], 13 RRMS	(245)
CXCL10	↑	31	–	31	–	25	6	19 Tx[g], 15 PDN, 7 IFN-β	29 RRMS, 18 ONND	(230)
	↑	17	3	16	4	19	1	2 SP, 5 PDN, 1 IFN-β, 1 IVIG	26 RRMS, 18 OND	(232)
	↑	13	–	13	–	NR	NR	–	15 ONND	(244)
	↔	8	2	NR	NR	NR	NR	9 Received Therapy[d]	10 MS, 6 LETM[e]	(184)
	↔	17	3	16	4	19	1	2 SP, 5 PDN, 1 IFN-β, 1 IVIG	9 PPMS	(232)
	↔	13	–	13	–	13	–	8 Immune Suppressed[i]	15 MS	(242)
	↔	13	–	13	–	NR	NR	–	17 MS	(244)
	↔	8	1	–	9	8	1	NR	8 L-NMOSD[j], 13 RRMS	(245)
CXCL12	↑	14	4	18	–	NR	NR	–	15 ONND, 17 i-ON	(247)
	↓	18[m]	2[m]	20	–	20	–	NR	20 NMOSD with Poor Recovery[n]	(246)
CXCL13	↑	16	6	22	–	NR	NR	–	18 RRMS, 12 ONND	(233)
	↑	8	1	9	–	6	3	3 Corticosteroids	9 ONND	(234)
	↑	29	8	37	–	NR	NR	PDN[f]	21 RRMS, 30 ONND	(252)
	↔	8	1	9	–	6	3	3 Corticosteroids	9 MS	(234)
CXCL14	↑	14	4	18	–	NR	NR	–	15 ONND, 17 i-ON	(247)
DBP	↓	16	9	25	–	NR	NR	–	30 OND	(187)
ECP	↑	6	2	8	–	NR	NR	–	16 RRMS, 16 SPMS, 16 HC	(66)
GDNF	↔	8	2	NR	NR	NR	NR	9 Received Therapy[d]	10 MS, 6 LETM[e]	(184)
Glial fibrillary acidic protein	↑	31	–	31	–	25	6	19 Tx[g], 15 PDN, 7 IFN-β	29 RRMS, 18 ONND	(230)
	↑	33	–	33	–	33	–	8 methyl-PDN	27 MS, 5 NB, 9 Meningitis, 12 OND	(237)
	↑	10	3	13	–	12	1	–	20 ONND, 9 i-ON, 9 i-Myelitis, 6 i-Encephalitis	(239)
	↑	18[m]	2[m]	20	–	20	–	NR	20 NMOSD with Poor Recovery[n]	(246)
	↑	10	–	10	–	10	–	NR	10 MS, 5 ONND, 3 ADEM, 3 SI	(249)
	↑	39	3	42	–	36	6	17 Tx[g], 14 PDN, 3 IFN-β, 5 AZA	30 RRMS, 30 ONND	(250)
Granulocyte colony stimulating factor	↑	31	–	31	–	25	6	19 Tx[g], 15 PDN, 7 IFN-β	29 RRMS, 18 ONND	(230)
	↑	17	3	16	4	19	1	2 SP, 5 PDN, 1 IFN-β, 1 IVIG	18 OND	(232)
	↔	8	2	NR	NR	NR	NR	9 Received Therapy[d]	10 MS, 6 LETM[e]	(184)
	↔	17	3	16	4	19	1	2 SP, 5 PDN, 1 IFN-β, 1 IVIG	26 RRMS, 9 PPMS	(232)
	↔	19[h]	1[h]	20	–	NR	NR	10 IFN-β or Corticosteroids	20 RRMS, 19 SCD	(235)
	↔	NR[hk]	NR[hk]	10	–	NR	NR	–	15 RRMS[l], 19 SCD	(235)
Granulocyte macrophage colony stimulating factor	↔	8	2	NR	NR	NR	NR	9 Received Therapy[d]	10 MS, 6 LETM[e]	(184)
	↔	31	–	31	–	25	6	19 Tx[g], 15 PDN, 7 IFN-β	18 ONND	(230)
	↔	17	3	16	4	19	1	2 SP, 5 PDN, 1 IFN-β, 1 IVIG	26 RRMS, 9 PPMS, 18 OND	(232)
	↓	31	–	31	–	25	6	19 Tx[g], 15 PDN, 7 IFN-β	29 RRMS	(230)
Haptoglobin	↑	22	3	25	–	NR	NR	NR	16 MS, 15 AD, 22 OND	(192)
	↑	16	9	25	–	NR	NR	–	30 OND	(187)
High mobility box protein 1	↑	39	3	42	–	36	6	17 Tx[g], 14 PDN, 3 IFN-β, 5 AZA	30 RRMS, 30 ONND	(250)
	↑	16	6	22	–	18	4	–	14 ONND	(251)
	↔	16	6	22	–	18	4	–	18 RRMS	(251)
IL-1β	↑	19[h]	1[h]	20	–	NR	NR	10 IFN-β or Corticosteroids	19 SCD	(235)
	↑	8	1	–	9	8	1	NR	8 L-NMOSD[j], 13 RRMS	(245)

	↔	8	2	NR	NR	NR	NR	9 Received Therapy[d]	10 MS, 6 LETM[e]	(184)
	↔	31	–	31	–	25	6	19 Tx[g], 15 PDN, 7 IFN-β	29 RRMS, 18 ONND	(230)
	↔	17	3	16	4	19	1	2 SP, 5 PDN, 1 IFN-β, 1 IVIG	26 RRMS, 9 PPMS, 18 OND	(232)
	↔	19[h]	1[h]	20	–	NR	NR	10 IFN-β or Corticosteroids	20 RRMS	(235)
	↔	NR[hk]	NR[hk]	10	–	NR	NR	–	15 RRMS[l], 19 SCD	(235)
	↔	27	2	29	–	24	5	15 Tx[g], 14 PDN, 1 IFN-β, 1 AZA	29 RRMS, 17 ONND, 10 OIND	(241)
IL-1Ra	↑	31	–	31	–	25	6	19 Tx[g], 15 PDN, 7 IFN-β	18 ONND	(230)
	↔	8	2	NR	NR	NR	NR	9 Received Therapy[d]	10 MS, 6 LETM[e]	(184)
	↔	31	–	31	–	25	6	19 Tx[g], 15 PDN, 7 IFN-β	29 RRMS	(230)
	↔	17	3	16	4	19	1	2 SP, 5 PDN, 1 IFN-β, 1 IVIG	26 RRMS, 9 PPMS, 18 OND	(232)
IL-2	↔	8	2	NR	NR	NR	NR	9 Received Therapy[d]	10 MS, 6 LETM[e]	(184)
	↔	17	–	17	–	17	–	2 IFN-β, 4 PDN, 1 PDN+CsA	21 RRMS, 21 OND	(228)
	↔	31	–	31	–	25	6	19 Tx[g], 15 PDN, 7 IFN-β	29 RRMS, 18 ONND	(230)
	↔	17	3	16	4	19	1	2 SP, 5 PDN, 1 IFN-β, 1 IVIG	26 RRMS, 9 PPMS, 18 OND	(232)
	↔	19[h]	1[h]	20	–	NR	NR	10 IFN-β or Corticosteroids	20 RRMS, 19 SCD	(235)
	↔	NR[hk]	NR[hk]	10	–	NR	NR	–	15 RRMS[l], 19 SCD	(235)
	↔	8	1	–	9	8	1	NR	8 L-NMOSD[j], 13 RRMS	(245)
IL-4	↔	8	2	NR	NR	NR	NR	9 Received Therapy[d]	10 MS, 6 LETM[e]	(184)
	↔	17	–	17	–	17	–	2 IFN-β, 4 PDN, 1 PDN+CsA	21 RRMS, 21 OND	(228)
	↔	31	–	31	–	25	6	19 Tx[g], 15 PDN, 7 IFN-β	29 RRMS, 18 ONND	(230)
	↔	17	3	16	4	19	1	2 SP, 5 PDN, 1 IFN-β, 1 IVIG	26 RRMS, 9 PPMS, 18 OND	(232)
	↔	19[h]	1[h]	20	–	NR	NR	10 IFN-β or Corticosteroids	20 RRMS, 19 SCD	(235)
	↔	NR[hk]	NR[hk]	10	–	NR	NR	–	15 RRMS[l], 19 SCD	(235)
	↔	27	2	29	–	24	5	15 Tx[g], 14 PDN, 1 IFN-β, 1 AZA	29 RRMS, 17 ONND, 10 OIND	(241)
	↔	8	1	–	9	8	1	NR	8 L-NMOSD[j], 13 RRMS	(245)
IL-5	↑	8	4	9	3	NR	NR	–	24 RRMS, 24 SPMS, 24 OIND, 24 HC	(214)
	↑	6	2	8	–	NR	NR	–	16 RRMS, 16 SPMS, 16 HC	(66)
	↑	19[h]	1[h]	20	–	NR	NR	10 IFN-β or Corticosteroids	20 RRMS	(235)
	↔	8	2	NR	NR	NR	NR	9 Received Therapy[d]	10 MS, 6 LETM[e]	(184)
	↔	31	–	31	–	25	6	19 Tx[g], 15 PDN, 7 IFN-β	29 RRMS, 18 ONND	(230)
	↔	17	3	16	4	19	1	2 SP, 5 PDN, 1 IFN-β, 1 IVIG	26 RRMS, 9 PPMS, 18 OND	(232)
	↔	19[h]	1[h]	20	–	NR	NR	10 IFN-β or Corticosteroids	19 SCD	(235)
	↔	NR[hk]	NR[hk]	10	–	NR	NR	–	15 RRMS[l], 19 SCD	(235)
	↔	8	1	–	9	8	1	NR	8 L-NMOSD[j], 13 RRMS	(245)
IL-6	↑	17	–	17	–	17	–	2 IFN-β, 4 PDN, 1 PDN+CsA	21 RRMS, 21 OND	(228)
	↑	8	4	9	3	NR	NR	–	24 RRMS, 24 SPMS, 24 OIND, 24 HC	(214)
	↑	6	2	8	–	NR	NR	–	16 RRMS, 16 SPMS, 16 HC	(66)
	↑	31	–	31	–	25	6	19 Tx[g], 15 PDN, 7 IFN-β	29 RRMS, 18 ONND	(230)
	↑	16	6	22	–	18	4	–	18 MS, 14 ONND	(231)
	↑	17	3	16	4	19	1	2 SP, 5 PDN, 1 IFN-β, 1 IVIG	26 RRMS, 9 PPMS, 18 OND	(232)
	↑	10	3	13	–	12	1	–	20 ONND, 9 i-ON, 9 i-Myelitis	(239)
	↑	50	9	59	–	NR	NR	–	76 MS, 70 OIND, 24 TM, 27 i-ON, 39 ONND, 45 NDD	(240)
	↑	27	2	29	–	24	5	15 Tx[g], 14 PDN, 1 IFN-β, 1 AZA	29 RRMS, 17 ONND, 10 OIND	(241)
	↑	8	1	–	9	8	1	NR	8 L-NMOSD[j], 13 RRMS	(245)
	↑	39	3	42	–	36	6	17 Tx[g], 14 PDN, 3 IFN-β, 5 AZA	30 RRMS, 30 ONND	(250)
	↑	16	6	22	–	18	4	–	18 RRMS, 14 ONND	(251)
	↑	28	2	30	–	26	4	NR	22 RRMS, 24 ONND	(254)
	↔	8	2	NR	NR	NR	NR	9 Received Therapy[d]	10 MS, 6 LETM[e]	(184)
	↔	19[h]	1[h]	20	–	NR	NR	10 IFN-β or Corticosteroids	20 RRMS, 19 SCD	(235)
	↔	NR[hk]	NR[hk]	10	–	NR	NR	–	15 RRMS[l], 19 SCD	(235)
	↔	10	3	13	–	12	1	–	6 i-Encephalitis	(239)
	↔	50	9	59	–	NR	NR	–	10 CNS VD, 15 CNS Tumors, 15 Epilepsy	(240)
IL-7	↔	8	2	NR	NR	NR	NR	9 Received Therapy[d]	10 MS, 6 LETM[e]	(184)
	↔	31	–	31	–	25	6	19 Tx[g], 15 PDN, 7 IFN-β	29 RRMS, 18 ONND	(230)
	↔	17	3	16	4	19	1	2 SP, 5 PDN, 1 IFN-β, 1 IVIG	26 RRMS, 9 PPMS, 18 OND	(232)

	↔	19[h]	1[h]	20	–	NR	NR	10 IFN-β or Corticosteroids	20 RRMS	(235)
	↔	NR[hk]	NR[hk]	10	–	NR	NR	–	15 RRMS[l], 19 SCD	(235)
	↓	19[h]	1[h]	20	–	NR	NR	10 IFN-β or Corticosteroids	19 SCD	(235)
IL-9	↔	8	2	NR	NR	NR	NR	9 Received Therapy[d]	10 MS, 6 LETM[e]	(184)
	↔	31	–	31	–	25	6	19 Tx[g], 15 PDN, 7 IFN-β	18 ONND	(230)
	↔	17	3	16	4	19	1	2 SP, 5 PDN, 1 IFN-β, 1 IVIG	26 RRMS, 9 PPMS, 18 OND	(232)
	↓	31	–	31	–	25	6	19 Tx[g], 15 PDN, 7 IFN-β	29 RRMS	(230)
IL-10	↑	31	–	31	–	25	6	19 Tx[g], 15 PDN, 7 IFN-β	18 ONND	(230)
	↑	19[h]	1[h]	20	–	NR	NR	10 IFN-β or Corticosteroids	19 SCD	(235)
	↔	8	2	NR	NR	NR	NR	9 Received Therapy[d]	10 MS, 6 LETM[e]	(184)
	↔	17	–	17	–	17	–	2 IFN-β, 4 PDN, 1 PDN+CsA	21 RRMS, 21 OND	(228)
	↔	31	–	31	–	25	6	19 Tx[g], 15 PDN, 7 IFN-β	29 RRMS	(230)
	↔	17	3	16	4	19	1	2 SP, 5 PDN, 1 IFN-β, 1 IVIG	26 RRMS, 9 PPMS, 18 OND	(232)
	↔	19[h]	1[h]	20	–	NR	NR	10 IFN-β or Corticosteroids	20 RRMS	(235)
	↔	NR[hk]	NR[hk]	10	–	NR	NR	–	15 RRMS[l], 19 SCD	(235)
	↔	27	2	29	–	24	5	15 Tx[g], 14 PDN, 1 IFN-β, 1 AZA	29 RRMS, 17 ONND, 10 OIND	(241)
	↔	8	1	–	9	8	1	NR	8 L-NMOSD[j], 13 RRMS	(245)
IL-12	↑	6	2	8	–	NR	NR	–	16 HC	(66)
	↔	8	2	NR	NR	NR	NR	9 Received Therapy[d]	10 MS, 6 LETM[e]	(184)
	↔	6	2	8	–	NR	NR	–	16 RRMS, 16 SPMS	(66)
	↔	31	–	31	–	25	6	19 Tx[g], 15 PDN, 7 IFN-β	29 RRMS, 18 ONND	(230)
	↔	17	3	16	4	19	1	2 SP, 5 PDN, 1 IFN-β, 1 IVIG	26 RRMS, 9 PPMS, 18 OND	(232)
	↔	19[h]	1[h]	20	–	NR	NR	10 IFN-β or Corticosteroids	20 RRMS, 19 SCD	(235)
	↔	NR[hk]	NR[hk]	10	–	NR	NR	–	15 RRMS[l], 19 SCD	(235)
	↔	8	1	–	9	8	1	NR	8 L-NMOSD[j], 13 RRMS	(245)
IL-13	↑	8	4	9	3	NR	NR	–	24 RRMS, 24 SPMS, 24 OIND, 24 HC	(214)
	↑	31	–	31	–	25	6	19 Tx[g], 15 PDN, 7 IFN-β	29 RRMS, 18 ONND	(230)
	↑	19[h]	1[h]	20	–	NR	NR	10 IFN-β or Corticosteroids	19 SCD	(235)
	↔	8	2	NR	NR	NR	NR	9 Received Therapy[d]	10 MS, 6 LETM[e]	(184)
	↔	17	3	16	4	19	1	2 SP, 5 PDN, 1 IFN-β, 1 IVIG	26 RRMS, 9 PPMS, 18 OND	(232)
	↔	19[h]	1[h]	20	–	NR	NR	10 IFN-β or Corticosteroids	20 RRMS	(235)
	↔	NR[hk]	NR[hk]	10	–	NR	NR	–	15 RRMS[l], 19 SCD	(235)
IL-15	↔	8	2	NR	NR	NR	NR	9 Received Therapy[d]	10 MS, 6 LETM[e]	(184)
	↔	31	–	31	–	25	6	19 Tx[g], 15 PDN, 7 IFN-β	29 RRMS, 18 ONND	(230)
	↔	17	3	16	4	19	1	2 SP, 5 PDN, 1 IFN-β, 1 IVIG	26 RRMS, 9 PPMS, 18 OND	(232)
IL-17	↑	19[h]	1[h]	20	–	NR	NR	10 IFN-β or Corticosteroids	20 RRMS, 19 SCD	(235)
	↑	NR[hk]	NR[hk]	10	–	NR	NR	–	15 RRMS[l], 19 SCD	(235)
	↔	31	–	31	–	25	6	19 Tx[g], 15 PDN, 7 IFN-β	29 RRMS, 18 ONND	(230)
	↔	13	–	13	–	13	–	8 Immune Suppressed[i]	15 MS	(242)
IL-17A	↑	17	3	16	4	19	1	2 SP, 5 PDN, 1 IFN-β, 1 IVIG	26 RRMS, 18 OND	(232)
	↑	16	6	22	–	18	4	–	18 RRMS, 14 ONND	(251)
	↔	8	2	NR	NR	NR	NR	9 Received Therapy[d]	10 MS, 6 LETM[e]	(184)
	↔	31	–	31	–	25	6	19 Tx[g], 15 PDN, 7 IFN-β	29 RRMS, 18 ONND	(230)
	↔	17	3	16	4	19	1	2 SP, 5 PDN, 1 IFN-β, 1 IVIG	9 PPMS	(232)
	↔	27	2	29	–	24	5	15 Tx[g], 14 PDN, 1 IFN-β, 1 AZA	17 ONND, 10 OIND	(241)
	↓	27	2	29	–	24	5	15 Tx[g], 14 PDN, 1 IFN-β, 1 AZA	29 RRMS	(241)
IL-17F	↔	27	2	29	–	24	5	15 Tx[g], 14 PDN, 1 IFN-β, 1 AZA	29 RRMS, 17 ONND, 10 OIND	(241)
IL-21	↑	16	5	21	–	21	–	–	16 ONND	(243)
	↔	27	2	29	–	24	5	15 Tx[g], 14 PDN, 1 IFN-β, 1 AZA	29 RRMS, 17 ONND, 10 OIND	(241)
	↔	16	5	21	–	21	–	–	20 RRMS	(243)
IL-22	↔	27	2	29	–	24	5	15 Tx[g], 14 PDN, 1 IFN-β, 1 AZA	29 RRMS, 17 ONND, 10 OIND	(241)
IL-23	↔	31	–	31	–	25	6	19 Tx[g], 15 PDN, 7 IFN-β	29 RRMS, 18 ONND	(230)
	↔	27	2	29	–	24	5	15 Tx[g], 14 PDN, 1 IFN-β, 1 AZA	29 RRMS, 17 ONND, 10 OIND	(241)
IL-25	↔	27	2	29	–	24	5	15 Tx[g], 14 PDN, 1 IFN-β, 1 AZA	29 RRMS, 17 ONND, 10 OIND	(241)
IL-27	↔	37	8	NR	NR	36	9	PDN,[f] AZA[f]	19 ONND, 40 HC	(253)

IL-31	↔	27	2	29	–	24	5	15 Tx[g], 14 PDN, 1 IFN-β, 1 AZA	29 RRMS, 17 ONND, 10 OIND	(241)
IL-33	↔	27	2	29	–	24	5	15 Tx[g], 14 PDN, 1 IFN-β, 1 AZA	29 RRMS, 17 ONND, 10 OIND	(241)
IL-35	↔	37	8	NR	NR	36	9	PDN,[f] AZA[f]	19 ONND, 40 HC	(253)
Interferon-γ	↔	8	2	NR	NR	NR	NR	9 Received Therapy[d]	10 MS, 6 LETM[e]	(184)
	↔	17	–	17	–	17	–	2 IFN-β, 4 PDN, 1 PDN+CsA	21 RRMS, 21 OND	(228)
	↔	31	–	31	–	25	6	19 Tx[g], 15 PDN, 7 IFN-β	29 RRMS, 18 ONND	(230)
	↔	17	3	16	4	19	1	2 SP, 5 PDN, 1 IFN-β, 1 IVIG	26 RRMS, 9 PPMS, 18 OND	(232)
	↔	19[h]	1[h]	20	–	NR	NR	10 IFN-β or Corticosteroids	20 RRMS, 19 SCD	(235)
	↔	NR[hk]	NR[hk]	10	–	NR	NR	–	15 RRMS[l], 19 SCD	(235)
	↔	27	2	29	–	24	5	15 Tx[g], 14 PDN, 1 IFN-β, 1 AZA	29 RRMS, 17 ONND, 10 OIND	(241)
	↔	8	1	–	9	8	1	NR	8 L-NMOSD[j], 13 RRMS	(245)
Maltose binding protein	↑	8	1	9	–	6	3	3 Corticosteroids	9 ONND	(234)
	↑	33	–	33	–	33	–	8 methyl-PDN	12 OND	(237)
	↔	8	1	9	–	6	3	3 Corticosteroids	9 MS	(234)
	↔	33	–	33	–	33	–	8 methyl-PDN	27 MS, 6 ADEM, 5 NB, 9 Meningitis, 3 SI	(237)
MMP-1	↔	27	2	29	–	24	5	15 Tx[g], 14 PDN, 1 IFN-β, 1 AZA	29 RRMS, 17 ONND, 10 OIND	(241)
MMP-2	↑	27	2	29	–	24	5	15 Tx[g], 14 PDN, 1 IFN-β, 1 AZA	29 RRMS, 17 ONND, 10 OIND	(241)
MMP-3	↔	27	2	29	–	24	5	15 Tx[g], 14 PDN, 1 IFN-β, 1 AZA	29 RRMS, 17 ONND, 10 OIND	(241)
MMP-4	↔	27	2	29	–	24	5	15 Tx[g], 14 PDN, 1 IFN-β, 1 AZA	29 RRMS, 17 ONND, 10 OIND	(241)
MMP-5	↔	27	2	29	–	24	5	15 Tx[g], 14 PDN, 1 IFN-β, 1 AZA	29 RRMS, 17 ONND, 10 OIND	(241)
MMP-6	↔	27	2	29	–	24	5	15 Tx[g], 14 PDN, 1 IFN-β, 1 AZA	29 RRMS, 17 ONND, 10 OIND	(241)
MMP-7	↔	27	2	29	–	24	5	15 Tx[g], 14 PDN, 1 IFN-β, 1 AZA	29 RRMS, 17 ONND, 10 OIND	(241)
MMP-8	↔	27	2	29	–	24	5	15 Tx[g], 14 PDN, 1 IFN-β, 1 AZA	29 RRMS, 17 ONND, 10 OIND	(241)
MMP-9	↔	27	2	29	–	24	5	15 Tx[g], 14 PDN, 1 IFN-β, 1 AZA	29 RRMS, 17 ONND, 10 OIND	(241)
	↔	13	–	13	–	13	–	8 Immune Suppressed[i]	15 MS	(242)
NF heavy chain	↑	8	1	9	–	6	3	3 Corticosteroids	9 MS, 9 ONND	(234)
	↑	16	6	22	–	NR	NR	–	14 ONND	(248)
	↔	33	–	33	–	33	–	8 methyl-PDN	27 MS, 12 OND, 6 ADEM, 5 NB, 9 Meningitis, 3 SI	(237)
	↔	16	6	22	–	NR	NR	–	18 RRMS	(248)
NF light chain	↑	16	6	22	–	NR	NR	–	18 RRMS, 14 ONND	(248)
Platelet derived growth factor-BB	↑	14	4	18	–	NR	NR	–	15 ONND	(247)
	↔	8	2	NR	NR	NR	NR	9 Received Therapy[d]	10 MS, 6 LETM[e]	(184)
	↔	31	–	31	–	25	6	19 Tx[g], 15 PDN, 7 IFN-β	29 RRMS, 18 ONND	(230)
	↔	17	3	16	4	19	1	2 SP, 5 PDN, 1 IFN-β, 1 IVIG	26 RRMS, 9 PPMS, 18 OND	(232)
	↔	14	4	18	–	NR	NR	–	17 i-ON	(247)
Resistin	↑	47	9	56	–	44	12	–	12 ONND	(255)
S100 calcium-binding protein B	↑	33	–	33	–	33	–	8 methyl-PDN	27 MS, 12 OND, 5 NB	(237)
	↑	10	–	10	–	10	–	NR	10 MS, 5 ONND, 3 ADEM, 3 SI	(249)
	↔	8	2	NR	NR	NR	NR	9 Received Therapy[d]	10 MS, 6 LETM[e]	(184)
	↔	33	–	33	–	33	–	8 methyl-PDN	6 ADEM, 9 Meningitis, 3 SI	(237)
sCD40L	↑	27	2	29	–	24	5	15 Tx[g], 1 AZA, 14 PDN, 1 IFN-β	10 OIND, 17 ONND	(72)
	↔	27	2	29	–	24	5	15 Tx[g], 1 AZA, 14 PDN, 1 IFN-β	29 MS	(72)
sICAM-1	↑	25	–	25	–	19	6	12 PDN	21 RRMS, 20 ONND	(238)
sIL-6R	↑	16	6	22	–	18	4	–	18 MS, 14 ONND	(231)
sRAGE	↔	8	2	NR	NR	NR	NR	9 Received Therapy[d]	10 MS, 6 LETM[e]	(184)
sVCAM-1	↑	25	–	25	–	19	6	12 PDN	21 RRMS, 20 ONND	(238)
TGF-β	↔	31	–	31	–	25	6	19 Tx[g], 15 PDN, 7 IFN-β	29 RRMS, 18 ONND	(230)
TIMP-1	↑	27	2	29	–	24	5	15 Tx[g], 14 PDN, 1 IFN-β, 1 AZA	29 RRMS, 17 ONND, 10 OIND	(241)
	↑	13	–	13	–	13	–	8 Immune Suppressed[i]	15 MS	(242)
TIMP-2	↔	27	2	29	–	24	5	15 Tx[g], 14 PDN, 1 IFN-β, 1 AZA	29 RRMS, 17 ONND, 10 OIND	(241)
TIMP-3	↔	27	2	29	–	24	5	15 Tx[g], 14 PDN, 1 IFN-β, 1 AZA	29 RRMS, 17 ONND, 10 OIND	(241)
TIMP-4	↔	27	2	29	–	24	5	15 Tx[g], 14 PDN, 1 IFN-β, 1 AZA	29 RRMS, 17 ONND, 10 OIND	(241)
	↑	19[h]	1[h]	20	–	NR	NR	10 IFN-β or Corticosteroids	20 SCD	(235)
	↔	8	2	NR	NR	NR	NR	9 Received Therapy[d]	10 MS, 6 LETM[e]	(184)

Tissue Necrosis Factor-α	↔	17	–	17	–	17	–	2 IFN-β, 4 PDN, 1 PDN+CsA	21 RRMS, 21 OND	(228)
	↔	31	–	31	–	25	6	19 Tx[g], 15 PDN, 7 IFN-β	18 ONND	(230)
	↔	17	3	16	4	19	1	2 SP, 5 PDN, 1 IFN-β, 1 IVIG	26 RRMS, 9 PPMS, 18 OND	(232)
	↔	19[h]	1[h]	20	–	NR	NR	10 IFN-β or Corticosteroids	20 RRMS	(235)
	↔	NR[hk]	NR[hk]	10	–	NR	NR	–	15 RRMS[l], 19 SCD	(235)
	↔	27	2	29	–	24	5	15 Tx[g], 14 PDN, 1 IFN-β, 1 AZA	29 RRMS, 17 ONND, 10 OIND	(241)
	↔	8	1	–	9	8	1	NR	8 L-NMOSD[j], 13 RRMS	(245)
	↔	18[m]	2[m]	20	–	20	–	NR	20 NMOSD with Poor Recovery[n]	(246)
	↓	31	–	31	–	25	6	19 Tx[g], 15 PDN, 7 IFN-β	29 RRMS	(230)
Vascular endothelial growth factor	↔	8	2	NR	NR	NR	NR	9 Received Therapy[d]	10 MS, 6 LETM[e]	(184)
	↔	31	–	31	–	25	6	19 Tx[g], 15 PDN, 7 IFN-β	18 ONND	(230)
	↔	17	3	16	4	19	1	2 SP, 5 PDN, 1 IFN-β, 1 IVIG	26 RRMS, 9 PPMS, 18 OND	(232)

ADEM, acute disseminated encephalomyelitis; AD, Alzheimer's disease; AMCase, acidic mammalian chitinase; APRIL, a proliferation-inducing ligand; AQP4-Ab, aquaporin-4 Antibody; AZA, azathioprine; C5b-9, terminal complement complex; CCL, chemokine (C-C motif) ligand; CHI3L1, chitinase-3-like protein 1; ChT, chitotriosidase; CNS, central nervous system; CNS VD, central nervous system vascular disorders; CsA, Cyclosporin A; CXCL, chemokine (C-X-C motif) ligand; DBP, vitamin D binding protein; ECP, eosinophil cationic protein; GDNF, glial cell line-derived neurotrophic factor; HC, healthy control; i-encephalitis, idiopathic encephalitis; IFN-β, interferon-β; IL, interleukin; IL-1Ra, interleukin-1 receptor antagonist; i-myelitis, idiopathic myelitis; i-ON, idiopathic optic neuritis; IVIG, intravenous immunoglobulin; LAP, relapse; LETM, longitudinally extensive transverse myelitis; L-NMOSD, limited neuromyelitis optica spectrum disorder; MBP, maltose binding protein; MMP, matrix metalloproteinase; MS, multiple sclerosis; NB, neuro-Behçet disease; NDD, neurodegenerative disease; NF, neurofilament; NMOSD, neuromyelitis optica spectrum disorder; NR, Not reported; OIND, other inflammatory neurologic disorders; ON, optic neuritis; OND, other neurologic disorders; ONND, other non-inflammatory neurologic disorders; PDN, prednisolone; PPMS, primary progressive multiple sclerosis; RRMS, relapsing remitting multiple sclerosis; SCD, spinocerebellar degeneration; sCD40L, soluble CD40 ligand; SI, spinal infarct; sICAM-1, soluble intercellular adhesion molecule 1; sIL-6R, soluble interleukin-6 receptor; SP, steroid pulse; SPMS, secondary progressive multiple sclerosis; sRAGE, soluble receptor for advanced glycation end-products; sVCAM-1, soluble vascular cell adhesion molecule 1; TGF-β, transforming growth factor-β; TIMP, tissue inhibitor of metalloproteinases; TM, transverse myelitis; Tx, treatment.

[a] *Differentiates between NMOSD patient disease status at time of biopsy sampling, not between monophasic vs. relapsing-remitting forms of disease.*
[b] *Defines preventative treatments provided during patient's remitting period of disease, not for treatment of acute relapse. Patients not receiving treatment during remission were omitted from this column.*
[c] *If multiple groups are listed, there was statistical significance reported between the study group and both comparison groups.*
[d] *Nine of the 10 NMOSD patients sampled were receiving therapeutic interventions prior to sampling, the specifics of which were not reported.*
[e] *Patients were seronegative for AQP4-IgG & MOG-IgG.*
[f] *The exact number and subset of patients taking medication was not reported.*
[g] *This study did not specify between patients receiving monotherapy or combination therapy.*
[h] *The study cohort was comprised of opticospinal MS patients, who have since been subclassified beneath the NMOSD spectrum.*
[i] *Patients received at least one immunomodulatory therapy, such as corticosteroids, azathioprine, methylprednisolone pulse therapy, or IFN-β.*
[j] *Limited NMOSD patients either had ON with AQP4-IgG seropositivity without brain, brainstem, or spinal cord lesions; or had myelitis with AQP4-IgG seropositivity without ON.*
[k] *This study cohort consisted of the 10 opticospinal MS patients who did not receive any immunomodulating therapies to treat their OSMS, however, the sex distribution of this study subgroup was not reported.*
[l] *Patients did not receive any immunomodulating therapies.*
[m] *Patients were diagnosed with NMOSD and had good recovery (defined as visual acuity score increasing at least 3 points) two months after an acute relapse with treatment on high dose methylprednisolone.*
[n] *Patients were diagnosed with NMOSD and had poor recovery (defined as visual acuity score increasing by less than 3 points) two months after an acute relapse with treatment on high dose methylprednisolone.*
