# Supplementary material for: Metabolomic Profiling in Neuromyelitis Optica Spectrum Disorder Biomarker Discovery
Source: Metabolites. 2020 Sep 18;10(9):374. doi: 10.3390/metabo10090374 (PMC7570337; doi:10.3390/metabo10090374)
Supplement: Supplementary file 1 [file metabolites-10-00374-s001.zip › Supplementary/Supplementary Table 3_NMOSD Manuscript_McKarns_10.07.19.pdf]

Supplementary Table 3. Sera Outcomes in Targeted Immunoassay-based NMOSD Studies.

Endpoint	Shift	NMOSD Cohort		Disease State at Biopsy[a]		AQP4-Ab Serology		Treatment[b]	Control[c]	Ref.
		F	M	LAP	REM	+	-			
Activated factor B	↔	22	6	28	–	15	13	–	29 RRMS, 31 HC	(222)
	↓	22	6	28	–	15	13	–	30 Behçet’s Disease	(222)
AMCase	↔	8	4	9	3	NR	NR	–	24 RRMS, 24 SPMS, 24 OIND, 24 HC	(214)
AOPP	↑	NR[d]	NR[d]	–	11	NR	NR	–	11 HC	(261)
BAFF	↑	7	3	10	–	NR	NR	–	10 RRMS, 10 ONND	(236)
	↔	22	2	–	24	NR	NR	7 GC, 2 AZA, 7 GC + AZA	20 RRMS, 22 HC	(266)
C3	↑	24[e]	1[e]	–	✓	25	–	NR	54 MS[f]	(224)
	↔	24[e]	1[e]	–	✓	25	–	NR	39 OSMS[f]	(224)
	↔	24[e]	1[e]	✓	–	25	–	NR	54 MS[f], 39 OSMS[f]	(224)
C3a	↔	15	–	15	–	15	–	NR	15 MS, 12 OND	(223)
C4	↔	24[e]	1[e]	–	✓	25	–	NR	54 MS[f], 39 OSMS[f]	(224)
	↔	24[e]	1[e]	✓	–	25	–	NR	54 MS[f], 39 OSMS[f]	(224)
C4a	↔	15	–	15	–	15	–	NR	15 MS, 12 OND	(223)
C4d	↑	22	6	28	–	15	13	–	29 RRMS, 31 HC	(222)
	↔	22	6	28	–	15	13	–	30 Behçet’s Disease	(222)
	↔	15	–	15	–	15	–	NR	15 MS, 12 OND	(223)
C5a	↔	15	–	15	–	15	–	NR	15 MS, 12 OND	(223)
C5b-9	↑	22	6	28	–	15	13	–	29 RRMS, 31 HC	(222)
	↓	22	6	28	–	15	13	–	30 Behçet’s Disease	(222)
Catalase	↑	NR[d]	NR[d]	–	11	NR	NR	–	11 HC	(261)
CCL2	↔	13	–	13	–	NR	NR	–	17 MS, 15 ONND	(244)
CCL4	↔	2	17	–	19	19	–	12 AZA, 13 PRN, 2 MMF, 1 CP, 1 RTX, 1 MTO	10 MS	(258)
CCL11	↑	4[g]	2[g]	–	6	4	2	2 Azathioprine, 1 Copaxone, 1 Interferon-β	10 NMOSD without LETM	(256)
	↔	13	–	13	–	NR	NR	–	17 MS, 15 ONND	(244)
	↔	2	17	–	19	19	–	12 AZA, 13 PRN, 2 MMF, 1 CP, 1 RTX, 1 MTO	10 MS	(258)
CCL17	↔	13	–	13	–	NR	NR	–	17 MS	(244)
	↓	13	–	13	–	NR	NR	–	15 ONND	(244)
CH50	↑	24[e]	1[e]	✓	–	25	–	NR	54 MS[f], 39 OSMS[f]	(224)
	↔	24[e]	1[e]	–	✓	25	–	NR	54 MS[f], 39 OSMS[f]	(224)
CHI3LI	↔	8	4	9	3	NR	NR	–	24 RRMS, 24 SPMS, 24 OIND, 24 HC	(214)
ChT	↔	8	4	9	3	NR	NR	–	24 RRMS, 24 SPMS, 24 OIND, 24 HC	(214)
CRP	↔	24[e]	1[e]	–	✓	25	–	NR	54 MS[f], 39 OSMS[f]	(224)
	↔	24[e]	1[e]	✓	–	25	–	NR	54 MS[f], 39 OSMS[f]	(224)
CXCL1	↑	NR[gh]	NR[gh]	NR[h]	NR[h]	NR[h]	NR[h]	NR[h]	10 RRMS	(256)
CXCL5	↑	4[g]	2[g]	–	6	4	2	2 Azathioprine, 1 Copaxone, 1 Interferon-β	10 NMOSD without LETM	(256)
	↑	NR[gh]	NR[gh]	NR[h]	NR[h]	NR[h]	NR[h]	NR[h]	10 RRMS	(256)
	↑	23	2	–	25	NR	NR	NR	25 MS, 20 HC	(259)
CXCL8	↑	4[g]	2[g]	–	6	4	2	2 Azathioprine, 1 Copaxone, 1 Interferon-β	10 NMOSD without LETM	(256)
	↑	NR[gh]	NR[gh]	NR[h]	NR[h]	NR[h]	NR[h]	NR[h]	10 RRMS	(256)
	↔	31	–	31	–	25	6	19 Tx[i], 15 Prednisolone, 7 Interferon-β	18 ONND	(230)
	↔	2	17	–	19	19	–	12 AZA, 13 PRN, 2 MMF, 1 CP, 1 RTX, 1 MTO	10 MS	(258)
CXCL10	↑	4[g]	2[g]	–	6	4	2	2 Azathioprine, 1 Copaxone, 1 Interferon-β	10 NMOSD without LETM	(256)
	↔	31	–	31	–	25	6	19 Any Tx[i], 15 Prednisolone, 7 Interferon-β	18 ONND	(230)
	↔	13	–	13	–	NR	NR	–	17 MS, 15 ONND	(244)
CXCL13	↑	8	1	9	–	6	3	3 Corticosteroids	9 ONND	(234)
	↑	22	2	–	24	NR	NR	7 GC, 2 AZA, 7 GC + AZA	22 HC	(266)
	↔	8	1	9	–	6	3	3 Corticosteroids	9 MS	(234)
	↔	22	2	–	24	NR	NR	7 GC, 2 AZA, 7 GC + AZA	20 RRMS	(266)
G-CSF	↔	2	17	–	19	19	–	12 AZA, 13 PRN, 2 MMF, 1 CP, 1 RTX, 1 MTO	10 MS	(258)
GFAP	↑	58	19	NR	NR	36[j]	27[j]	NR	48 RION, 39 UCON, 38 MSON, 18 CRION, 11 ION	(265)
	↔	58	19	NR	NR	36[i]	27[i]	NR	47 RRMS, 45 HC	(265)
Haptoglobin	↔	22	3	25	–	NR	NR	NR	16 MS, 15 AD, 22 OND	(192)

HMGB1	↔	39	3	42	–	36	6	17 Tx[i], 14 Prednisolone, 3 Interferon-β, 5 AZA	30 RRMS, 30 ONND	(250)
hs-CRP	↑	47	9	56	–	44	12	–	56 HC	(255)
IL-1β	↑	23	2	–	25	NR	NR	NR	20 HC	(259)
	↔	2	17	–	19	19	–	12 AZA, 13 PRN, 2 MMF, 1 CP, 1 RTX, 1 MTO	10 MS	(258)
	↔	23	2	–	25	NR	NR	NR	25 MS	(259)
IL-1Ra	↔	31	–	31	–	25	6	19 Tx[i], 15 Prednisolone, 7 Interferon-β	18 ONND	(230)
	↔	2	17	–	19	19	–	12 AZA, 13 PRN, 2 MMF, 1 CP, 1 RTX, 1 MTO	10 MS	(258)
IL-2	↑	32	2	–	34	14	19	20 Interferon-β	24 RRMS, 30 HC	(264)
IL-4	↑	21	7	NR	NR	NR	NR	28 Methylprednisolone	28 HC	(260)
	↑	32	2	–	34	14	19	20 Interferon-β	24 RRMS, 30 HC	(264)
	↔	2	17	–	19	19	–	12 AZA, 13 PRN, 2 MMF, 1 CP, 1 RTX, 1 MTO	10 MS	(258)
IL-5	↔	4[g]	2[g]	–	6	4	2	2 Azathioprine, 1 Copaxone, 1 Interferon-β	10 NMOSD without LETM	(256)
IL-6	↑	31	–	31	–	25	6	19 Tx[i], 15 Prednisolone, 7 Interferon-β	18 ONND	(230)
	↑	32	2	–	34	14	19	20 Interferon-β	30 HC	(264)
	↔	27	2	29	–	24	5	15 Tx[i], 14 Prednisolone, 1 Interferon-β, 1 AZA	29 RRMS, 17 ONND, 10 OIND	(241)
	↔	10	4	14	–	11	3	–	20 RRMS, 16 HC	(257)
	↔	2	17	–	19	19	–	12 AZA, 13 PRN, 2 MMF, 1 CP, 1 RTX, 1 MTO	10 MS	(258)
	↔	17	4	21	–	18	3	–	15 RRMS, 12 HC	(263)
	↔	32	2	–	34	14	19	20 Interferon-β	24 RRMS	(264)
IL-9	↔	2	17	–	19	19	–	12 AZA, 13 PRN, 2 MMF, 1 CP, 1 RTX, 1 MTO	10 MS	(258)
IL-10	↑	32	2	–	34	14	19	20 Interferon-β	24 RRMS, 30 HC	(264)
	↔	31	–	31	–	25	6	19 Tx[i], 15 Prednisolone, 7 Interferon-β	18 ONND	(230)
	↔	2	17	–	19	19	–	12 AZA, 13 PRN, 2 MMF, 1 CP, 1 RTX, 1 MTO	10 MS	(258)
	↓	NR[d]	NR[d]	–	11	NR	NR	–	11 HC	(261)
IL-12	↔	4[g]	2[g]	–	6	4	2	2 Azathioprine, 1 Copaxone, 1 Interferon-β	10 NMOSD without LETM	(256)
IL-12p40	↔	4[g]	2[g]	–	6	4	2	2 Azathioprine, 1 Copaxone, 1 Interferon-β	10 NMOSD without LETM	(256)
IL-13	↔	31	–	31	–	25	6	19 Tx[i], 15 Prednisolone, 7 Interferon-β	18 ONND	(230)
	↔	2	17	–	19	19	–	12 AZA, 13 PRN, 2 MMF, 1 CP, 1 RTX, 1 MTO	10 MS	(258)
IL-17	↑	32	2	–	34	14	19	20 Interferon-β	30 HC	(264)
	↔	32	2	–	34	14	19	20 Interferon-β	24 RRMS	(264)
IL-17A	↑	4[g]	2[g]	–	6	4	2	2 Azathioprine, 1 Copaxone, 1 Interferon-β	10 NMOSD without LETM	(256)
	↑	10	4	14	–	11	3	–	20 RRMS, 16 HC	(257)
	↑	2	17	–	19	19	–	12 AZA, 13 PRN, 2 MMF, 1 CP, 1 RTX, 1 MTO	10 MS	(258)
	↑	10[k]	5[k]	16	–	12	4	–	16 ONND	(262)
	↔	27	2	29	–	24	5	15 Tx[i], 14 Prednisolone, 1 Interferon-β, 1 AZA	29 RRMS, 17 ONND, 10 OIND	(241)
	↔	10[k]	5[k]	16	–	12	4	–	14 RRMS	(262)
IL-17F	↑	4[g]	2[g]	–	6	4	2	2 Azathioprine, 1 Copaxone, 1 Interferon-β	10 NMOSD without LETM	(256)
	↔	2	17	–	19	19	–	12 AZA, 13 PRN, 2 MMF, 1 CP, 1 RTX, 1 MTO	10 MS	(258)
IL-21	↑	10	4	14	–	11	3	–	16 HC	(257)
	↑	17	4	21	–	18	3	–	12 HC	(263)
	↔	10	4	14	–	11	3	–	20 RRMS	(257)
	↔	17	4	21	–	18	3	–	15 RRMS	(263)
IL-22	↑	17	4	21	–	18	3	–	12 HC	(263)
	↔	17	4	21	–	18	3	–	15 RRMS	(263)
IL-23	↑	10	4	14	–	11	3	–	16 HC	(257)
	↑	10[k]	5[k]	16	–	12	4	–	16 ONND	(262)
	↔	10	4	14	–	11	3	–	20 RRMS	(257)
	↔	10[k]	5[k]	16	–	12	4	–	14 RRMS	(262)
IL-27	↔	17	4	21	–	18	3	–	15 RRMS, 12 HC	(263)
	↓	37	8	NR	NR	36	9	Prednisolone[f], Azathioprine[f]	19 ONND, 40 HC	(253)
IL-35	↓	37	8	NR	NR	36	9	Prednisolone[f], Azathioprine[f]	19 ONND, 40 HC	(253)
Interferon-β	↑	4[g]	2[g]	–	6	4	2	2 Azathioprine, 1 Copaxone, 1 Interferon-β	10 NMOSD without LETM	(256)
Interferon-γ	↑	32	2	–	34	14	19	20 Interferon-β	24 RRMS, 30 HC	(264)
	↔	21	7	NR	NR	NR	NR	28 Methylprednisolone	28 HC	(260)
	↔	NR[d]	NR[d]	–	11	NR	NR	–	11 HC	(261)

	↔	17	4	21	–	18	3	–	12 HC	(263)
	↓	17	4	21	–	18	3	–	15 RRMS	(263)
MDA	↑	NR[d]	NR[d]	–	11	NR	NR	–	11 HC	(261)
MMP-2	↔	27	2	29	–	24	5	15 Tx[i], 14 Prednisolone, 1 Interferon-β, 1 AZA	29 RRMS, 17 ONND, 10 OIND	(241)
MMP-9	↑	13	–	13	–	13	–	8 Immune Suppressed[l]	15 MS	(242)
	↔	27	2	29	–	24	5	15 Tx[i], 14 Prednisolone, 1 Interferon-β, 1 AZA	29 RRMS, 17 ONND, 10 OIND	(241)
	↔	NR[d]	NR[d]	–	11	NR	NR	–	11 HC	(261)
MMP-13	↔	27	2	29	–	24	5	15 Tx[i], 14 Prednisolone, 1 Interferon-β, 1 AZA	29 RRMS, 17 ONND, 10 OIND	(241)
MPO	↔	2	17	–	19	19	–	12 AZA, 13 PRN, 2 MMF, 1 CP, 1 RTX, 1 MTO	10 MS	(258)
PTX-2	↔	2	17	–	19	19	–	12 AZA, 13 PRN, 2 MMF, 1 CP, 1 RTX, 1 MTO	10 MS	(258)
Resistin	↑	47	9	56	–	44	12	–	56 HC	(255)
S100B	↔	58	19	NR	NR	36[j]	27[j]	NR	48 RION, 39 UCON, 38 MSON, 18 CRION, 11 ION, 47 RRMS, 45 HC	(265)
sICAM-1	↑	25	–	25	–	19	6	12 Prednisolone	17 HC	(238)
	↔	25	–	25	–	19	6	12 Prednisolone	21 RRMS, 20 ONND	(238)
SOD	↑	NR[d]	NR[d]	–	11	NR	NR	–	11 HC	(261)
Soluble CD40 Ligand	↑	25	2	27	–	25	2	3 Corticosteroids + Azathioprine	14 HC	(267)
	↔	27	2	29	–	24	5	15 Tx[g], 14 Prednisolone, 1 Interferon-β, 1 AZA	29 MS, 17 ONND, 10 OIND	(72)
	↔	25	2	27	–	25	2	3 Corticosteroids + Azathioprine	19 RRMS	(267)
sVCAM-1	↔	25	–	25	–	19	6	12 Prednisolone	21 RRMS, 20 ONND, 17 HC	(238)
TIMP-1	↔	27	2	29	–	24	5	15 Tx[i], 14 Prednisolone, 1 Interferon-β, 1 AZA	29 RRMS, 17 ONND, 10 OIND	(241)
	↔	NR[d]	NR[d]	–	11	NR	NR	–	11 HC	(261)
TGF-β	↔	10	4	14	–	11	3	–	20 RRMS, 16 HC	(257)
THP	↑	NR[d]	NR[d]	–	11	NR	NR	–	11 HC	(261)
Tissue necrosis factor-α	↑	23	2	–	25	NR	NR	NR	20 HC	(259)
	↑	32	2	–	34	14	19	20 Interferon-β	30 HC	(264)
	↔	2	17	–	19	19	–	12 AZA, 13 PRN, 2 MMF, 1 CP, 1 RTX, 1 MTO	10 MS	(258)
	↔	23	2	–	25	NR	NR	NR	25 MS	(259)
	↔	32	2	–	34	14	19	20 Interferon-β	24 RRMS	(264)
	↓	NR[d]	NR[d]	–	11	NR	NR	–	11 HC	(261)

AMCase, acidic mammalian chitinase; AD, Alzheimer's disease; AOPP, advanced oxidation protein products; AQP4-Ab, aquaporin-4 autoantibody; AZA, azathioprine; BAFF, B-cell activating factor; C5b-9, terminal complement complex; CCL, chemokine (C-C motif) ligand; CHI3L1, chitinase-3-like proteins 1; ChT, chitotriosidase; CNS, central nervous system; CP, cyclophosphamide; CRION, chronic relapsing isolated optic neuritis; CRP, c-reactive protein; CXCL, chemokine (C-X-C motif) ligand; GC, glucocorticoids; G-CSF, granulocyte colony stimulating factor; GFAP, glial fibrillary acidic protein; HC, healthy control; HGMB1, high mobility box protein 1; hs-CRP, high sensitivity c-reactive protein; IL, interleukin; IL-1Ra, interleukin-1 receptor antagonist; ION, isolated optic neuritis; LAP, relapse; LETM, longitudinally extensive transverse myelitis; MDA, malonyldialdehyde; MMF, mycophenolate mofetil; MMP, matrix metalloproteinase; MPO, myeloperoxidase; MS, multiple sclerosis; MSON, multiple sclerosis optic neuritis; MTO, mitoxantrone; NMOSD, neuromyelitis optica spectrum disorder; NR, Not reported; OIND, other inflammatory neurologic disorders; ON, optic neuritis; OND, other neurologic disorders; ONND, other non-inflammatory neurologic disorders; PDN, prednisolone; PTX, pentraxin; RION, relapsing inflammatory optic neuropathy; REM, remission; RRMS, relapsing remitting multiple sclerosis; RTX, rituximab; S100B, S100 calcium-binding protein B; sICAM-1, soluble intercellular adhesion molecule 1; SOD, superoxide dismutase; SPMS, secondary progressive MS; sVCAM-1, soluble vascular cell adhesion molecule 1; TARC, thymus and activation regulated chemokine; TGF-β, transforming growth factor beta; THP, total hydroperoxides; TIMP, tissue inhibitor of metalloproteinases; TNF-α, tissue necrosis factor alpha; Tx, treatment; UCON, unclassified optic neuritis.

✓ Endpoint variation only applies to the disease state marked with the check mark, though the sample size was not explicitly reported.
[a] Differentiates between NMOSD patient disease status at time of biopsy sampling, not between monophasic vs. relapsing-remitting forms of disease.
[b] Defines preventative treatments provided during patient's remitting period of disease, not for treatment of acute relapse. Patients not receiving treatment during remission were omitted from this column.
[c] If multiple groups are listed, there was statistical significance reported between the study group and both comparison groups.
[d] This study cohort consisted of 11 relapsing-remitting NMOSD patients, however, the sex distribution of this study subgroup was not reported.
[e] This study cohort consisted of 25 AQP4-IgG seropositive MS patients, 19 of which fulfilled the revised 2006 Wingerchuk NMO diagnostic criteria, though the sex distribution of this subset of patients was not reported.
[f] Patients were seronegative for AQP4-IgG.
[g] NMOSD patients in study group presented with LETM, defined as spinal cord lesions extending continuously for more than 3 vertebral segments on MRI.
[h] One of the original six NMOSD patients was removed from the study prior to analysis of this endpoint, thereby altering the sample's demographic data.
[i] This study did not specify between patients receiving monotherapy or combination therapy.
[j] Though 63 NMOSD patients were tested for AQP4-IgG, 14 remained untested and therefore have an unknown autoantibody serostatus.
[k] Though this study reported an NMOSD study group with an n of 16, the sex distribution of only 15 patients were included in the demographic data.
[l] Patients received at least one immunomodulatory therapy, such as corticosteroids, azathioprine, methylprednisolone pulse therapy, or IFN-β.
