# Supplementary material for: Metabolomic Profiling in Neuromyelitis Optica Spectrum Disorder Biomarker Discovery
Source: Metabolites. 2020 Sep 18;10(9):374. doi: 10.3390/metabo10090374 (PMC7570337; doi:10.3390/metabo10090374)
Supplement: Supplementary file 1 [file metabolites-10-00374-s001.zip › Supplementary/Supplementary Table 4_NMOSD Manuscript_McKarns_10.07.19.pdf]

Supplementary Table 4. Plasma Outcomes in Targeted Immunoassay-based NMOSD Studies.

Endpoint	Shift	NMOSD Cohort		Disease State at Biopsy[a]		AQP4-Ab Serology		Treatment[b]	Control[c]	Ref.
		F	M	LAP	REM	+	-			
Bb	↑	46	8	9	45	39	15[d]	20 DMT, 8 Prednisolone, 16 DMT + Prednisolone	40 RRMS, 69 HC	(216)
Complement C1inh	↑	46	8	9	45	39	15[d]	20 DMT, 8 Prednisolone, 16 DMT + Prednisolone	40 RRMS, 69 HC	(216)
Complement C1s	↑	46	8	9	45	39	15[d]	20 DMT, 8 Prednisolone, 16 DMT + Prednisolone	40 RRMS, 69 HC	(216)
Complement C3	↓	37	2	9	45	39[e]	–	17 DMT, 4 Prednisolone, 10 DMT + Prednisolone	40 RRMS, 69 HC	(216)
Complement C3a	↑	15	4	10	9	19	–	10 Monotherapy[f], 6 Combination Tx[g], 1 Stem Cells	40 HC	(221)
	↔	15	4	10	9	19	–	10 Monotherapy[f], 6 Combination Tx[g], 1 Stem Cells	35 MS	(221)
Complement iC3b	↑	46	8	9	45	39	15[d]	20 DMT, 8 Prednisolone, 16 DMT + Prednisolone	40 RRMS, 69 HC	(216)
Complement C4a	↑	15	4	10	9	19	–	10 Monotherapy[f], 6 Combination Tx[g], 1 Stem Cells	35 MS, 40 HC	(221)
Complement C4d	↑	46	8	9	45	39	15[d]	20 DMT, 8 Prednisolone, 16 DMT + Prednisolone	40 RRMS, 69 HC	(216)
Complement C5	↑	46	8	9	45	39	15[d]	20 DMT, 8 Prednisolone, 16 DMT + Prednisolone	40 RRMS, 69 HC	(216)
Complement C5a	↑	46	8	9	45	39	15[d]	20 DMT, 8 Prednisolone, 16 DMT + Prednisolone	40 RRMS, 69 HC	(216)
Factor H	↑	46	8	9	45	39	15[d]	20 DMT, 8 Prednisolone, 16 DMT + Prednisolone	40 RRMS, 69 HC	(216)
HMGB1	↑	28	1	29	–	10	19	29 Azathioprine	20 RRMS	(271)
IL-1β	↑	16	4	–	20	15	5	–	20 HC	(269)
IL-6	↑	17	9	26	–	20	6	22 Low Dose Steroids, 18 Azathioprine	22 HC	(270)
	↑	16	4	–	20	15	5	–	20 HC	(269)
	↔	17	9	26	–	20	6	22 Low Dose Steroids, 18 Azathioprine	23 RRMS	(270)
IL-10	↔	16	4	–	20	15	5	–	20 HC	(269)
IL-12	↔	16	4	–	20	15	5	–	20 HC	(270)
IL-17	↑	28	1	29	–	10	19	29 Azathioprine	20 RRMS	(271)
	↑	17	9	26	–	20	6	22 Low Dose Steroids, 18 Azathioprine	22 HC	(270)
	↔	16	4	–	20	15	5	–	20 HC	(269)
	↔	17	9	26	–	20	6	22 Low Dose Steroids, 18 Azathioprine	23 RRMS	(270)
IL-21	↔	16	4	–	20	15	5	–	20 HC	(269)
IL-23	↔	16	4	–	20	15	5	–	20 HC	(269)
IL-32α	↑	17	9	26	–	20	6	22 Low Dose Steroids, 18 Azathioprine	23 RRMS, 22 HC	(270)
Interferon-γ	↑	28	1	29	–	10	19	29 Azathioprine	20 RRMS	(271)
Neutrophil Elastase	↑	4	2	–	6	5	1	3 Rituximab, 1 Plasma Exchange	7 HC	(256)
sC5b-9	↑	46	8	9	45	39	15[d]	20 DMT, 8 Prednisolone, 16 DMT + Prednisolone	40 RRMS, 69 HC	(216)
	↑	15	4	10	9	19	–	10 Monotherapy[f], 6 Combination Tx[g], 1 Stem Cells	40 HC	(221)
	↔	15	4	10	9	19	–	10 Monotherapy[f], 6 Combination Tx[g], 1 Stem Cells	35 MS	(221)
Tissue necrosis factor-α	↑	28	1	29	–	10	19	29 Azathioprine	20 RRMS	(271)
	↔	16	4	–	20	15	5	–	20 HC	(269)

AQP4-Ab, aquaporin-4 autoantibody; Bb, activated complement factor B; C1inh, C1-inhibitor; DMT, disease modifying therapies (including methotrexate, azathioprine, mycophenolate mofetil, rituximab, or intravenous immunoglobulin); HC, healthy controls; HMGB1, high mobility group box 1 protein; IL, interleukin; LAP, relapse; MS, multiple sclerosis; NMOSD, neuromyelitis optica spectrum disorder; NR, not reported; REM, remission; RRMS, relapsing-remitting multiple sclerosis; sC5b-9, soluble terminal complement complex; Tx, treatment.

[a] *Differentiates between NMOSD patient disease status at time of biopsy sampling, not between monophasic vs. relapsing-remitting forms of disease.*
[b] *Defines preventative treatments provided during patient's remitting period of disease, not for treatment of acute relapse. Patients not receiving treatment during remission were omitted from this column.*
[c] *If multiple groups are listed, there was statistical significance reported between the study group and both comparison groups.*
[d] *Seven of 15 AQP4-IgG negative NMOSD patients were MOG-IgG seropositive.*
[e] *C3 levels were significantly lower only in the AQP4-IgG positive NMOSD group compared to RRMS & HC groups.*
[f] *Monotherapies include low dose prednisone, mycophenolate mofetil, cyclophosphamide, and rituximab.*
[g] *Combination therapies could be widely variable, but must include two or more treatments, e.g. prednisone with azathioprine.*
