# Supplementary material for: Metabolomic Profiling in Neuromyelitis Optica Spectrum Disorder Biomarker Discovery
Source: Metabolites. 2020 Sep 18;10(9):374. doi: 10.3390/metabo10090374 (PMC7570337; doi:10.3390/metabo10090374)
Supplement: Supplementary file 1 [file metabolites-10-00374-s001.zip › Supplementary/Supplementary Table 5_NMOSD Manuscript_McKarns_10.07.19.pdf]

Supplementary Table 5. Peripheral Blood Outcomes in Targeted Immunoassay-based NMOSD Studies.

Endpoint	Shift	NMOSD Cohort		Disease State at Biopsy[a]		AQP4-Ab Serology		Treatment[b]	Control[c]	Ref.
		F	M	LAP	REM	+	-			
B-cell activating factor receptor	↑	39	12	51	–	29	22	7 Prednisone	37 HC	(252)
	↔	39	12	51	–	29	22	7 Prednisone	42 RRMS	(252)
Chemokine (C-C motif) ligand 24	↔	8	4	9	3	NR	NR	–	24 RRMS, 24 SPMS, 24 OIND, 24 HC	(214)
	↔	6	2	8	–	NR	NR	–	16 RRMS, 16 SPMS, 16 HC	(66)
Chemokine (C-C motif) ligand 26	↔	8	4	9	3	NR	NR	–	24 RRMS, 24 SPMS, 24 OIND, 24 HC	(214)
	↔	6	2	8	–	NR	NR	–	16 RRMS, 16 SPMS, 16 HC	(66)
C-X-C motif chemokine receptor 5	↑	39	12	51	–	29	22	7 Prednisone	37 HC	(252)
	↔	39	12	51	–	29	22	7 Prednisone	42 RRMS	(252)
Eosinophil cationic protein	↔	6	2	8	–	NR	NR	–	16 RRMS, 16 SPMS, 16 HC	(66)
IL-5	↔	8	4	9	3	NR	NR	–	24 RRMS, 24 SPMS, 24 OIND, 24 HC	(214)
	↔	6	2	8	–	NR	NR	–	16 RRMS, 16 SPMS, 16 HC	(66)
IL-6	↑	6	2	8	–	NR	NR	–	16 RRMS, 16 SPMS, 16 HC	(66)
	↔	8	4	9	3	NR	NR	–	24 RRMS, 24 SPMS, 24 OIND, 24 HC	(214)
IL-10	↓	39	12	51	–	29	22	7 Prednisone	42 RRMS, 37 HC	(252)
IL-12	↑	6	2	8	–	NR	NR	–	16 HC	(66)
	↔	6	2	8	–	NR	NR	–	16 RRMS, 16 SPMS	(66)
IL-13	↔	8	4	9	3	NR	NR	–	24 RRMS, 24 SPMS, 24 OIND, 24 HC	(214)
Interferon-γ	↑	39	12	51	–	29	22	7 Prednisone	37 HC	(252)
	↔	39	12	51	–	29	22	7 Prednisone	42 RRMS	(252)
Very late antigen 4	↔	39	12	51	–	29	22	7 Prednisone	42 RRMS, 37 HC	(252)

AQP4-Ab, aquaporin-4 autoantibody; HC, healthy controls; IL, interleukin; LAP, relapse; MS, multiple sclerosis; NMOSD, neuromyelitis optica spectrum disorder; OIND, other inflammatory neurologic disorders; NMOSD, neuromyelitis optica spectrum disorder; NR, not reported; REM, remission; RRMS, relapsing-remitting multiple sclerosis; SPMS, secondary progressive multiple sclerosis.

[a] Differentiates between NMOSD patient disease status at time of biopsy sampling, not between monophasic vs. relapsing-remitting forms of disease.

[b] Defines preventative treatments provided during patient's remitting period of disease, not for treatment of acute relapse. Patients not receiving treatment during remission were omitted from this column.

[c] If multiple groups are listed, there was statistical significance reported between the study group and both comparison groups.
